# Association between dental fear and eating disorders and Body Mass Index among Finnish university students: a national survey

**DOI:** 10.1186/s12903-021-01449-8

**Published:** 2021-03-04

**Authors:** Mohammad Jalil Sharifian, Vesa Pohjola, Kristina Kunttu, Jorma I. Virtanen

**Affiliations:** 1 Medical Research Centre, Oulu University Hospital, Oulu, Finland; 2 Department of Clinical Dentistry, University of Bergen, Bergen, Norway; 3 Unit of Oral Health Sciences, University of Oulu, Oulu, Finland; 4 Finnish Student Health Service, Helsinki, Finland; 5 Institute of Dentistry, University of Turku, Turku, Finland

**Keywords:** Dental fear, Eating disorders, SCOFF, BMI, Students

## Abstract

**Background:**

Little is known about the association between eating disorders (ED) and dental fear. This study investigated the association between dental fear and EDs through body mass index (BMI), and SCOFF (sick, control, one stone, fat, food) questionnaire among Finnish university students. We hypothesised that dental fear is associated with EDs and BMI.

**Methods:**

We used the latest data from the Finnish University Student Health Survey 2016. This survey targeted undergraduate Finnish students (n = 10,000) of academic universities and universities of applied sciences. We enquired about e.g. age, gender, height, weight, educational sector and perceived mental well-being. We used the SCOFF questionnaire to assess those at risk for developing EDs. The question ‘Do you feel scared about dental care?’ enquired about dental fear. We used the chi-square test and gender-specific logistic regression to analyse the associations between dental fear, EDs and BMI controlling for age, educational sector and mental well-being.

**Results:**

In total, 3110 students participated in the study. Overall 7.2% of the students reported high dental fear and 9.2% scored SCOFF positive; more women than men reported high dental fear (11.2% vs. 3.8%, p < 0.001) and scored positive on SCOFF (14.2% vs. 3.6%, p < 0.001). Gender modified the association between dental fear and EDs and BMI. Among females, when controlling for educational sector and BMI, those with positive SCOFF score were more likely to have high dental fear than those with negative SCOFF score (OR = 1.6; CI = 1.0–2.4). After adding perceived mental well-being to the gender-specific regression analyses, overweight and obese males, BMI ≥ 25 (OR = 2.4; CI 1.3–4.4) and females with poor to moderate mental well-being (OR = 2.1; CI 1.4–2.9) were more likely than their counterparts to have high dental fear.

**Conclusions:**

Among the Finnish university students BMI in males and problems of mental well-being in females were positively associated with high dental fear. The results of this study support possible common vulnerability factors that dental fear and other psychological disorders may share.

## Background

High dental fear with irregular dental attendance has long been a widespread dental health topic worldwide. Studies have reported that 12% to 29% of children and adolescents experience dental fear/anxiety [1] and that up to 37% of the adult population suffer from moderate or very high dental fear [2]. Despite advancements in new technologies in dentistry which have made treatment visits shorter and more comfortable, new studies report minor positive changes. Longitudinal studies have also shown that high dental fear persists regardless of age, gender or education level [3, 4].

Eating disorders (ED) are “mental disorders defined by abnormal eating habits that negatively affect a person’s physical or mental health” [5], of which Anorexia nervosa (AN), Bulimia nervosa (BN), and atypical EDs are the three main types. Eating disorders often occur together with other mental disorders. A study in Finland found that more than two thirds of ED patients presented with at least one other mental disorder [6]. Other studies have found that EDs and dental fear are associated with vulnerability to psychopathology [6, 7], suggesting that EDs and dental fear may share common underlying vulnerability factors. The cognitive vulnerability model provides ways of understanding the factors that contribute to the onset and maintenance of psychological problems [8].

Patients with high dental fear are more likely to present with psychological problems such as anxiety disorders, mood disorders, emotional dysregulation, alcohol dependence and regular tobacco use [9–11]. The same underlying factors are also prominent in people with eating disorders [12, 13]. Moreover, studies report higher levels of dental fear in EDs patients than in the general population [14, 15].

Like high dental fear, EDs are noticeably more prevalent among young women than among men [16]. In Finland, EDs reportedly occur in as many as 24% of adolescent girls [17] with the risk for bulimia nervosa peaking in the 16–20 years age group [18]. The late teens and early adulthood are considered a critical period for establishing habits and beliefs [19]. This transitional period of experimentation towards an independent lifestyle could affect their health behaviours, namely their eating habits [20]. Thus, using simple and valid screening tools such as SCOFF-questionnaire (Sick, Control, One stone, Fat, Food) is crucial for the early identification of potential patients developing EDs [21].

Concerns about body shape and weight play important roles in the development of EDs, which are evident in the close association of EDs with body mass index (BMI) [22]. Researchers often use this measure of weight to identify patterns of EDs [23, 24]. Few studies have investigated the association between dental fear and weight (BMI), and the results are inconsistent [25, 26].

Patients with high dental fear or ED present with major dental health issues [11]. However, studies reporting an association between dental fear and EDs are scarce. This study aimed to evaluate the associations between dental fear and EDs (SCOFF status) and BMI, while controlling for age, gender, educational sector, attitude to food, and mental well-being among a representative sample of Finnish university students. We hypothesised that high dental fear is associated with EDs and BMI; those with risk for EDs or abnormal BMI are more prone to high dental fear compared to those without risk for EDs or normal BMI.

## Methods

### Study design and settings

The present cross-sectional study used the data from a national survey conducted every four years by the Finnish Student Health Service (FSHS) known as the national University Student Health Survey (USHS). The USHS aims to investigate students’ physical, mental and social health, key aspects of health-related behaviour, as well as the use of health services and opinions concerning the quality of the services [27]. In addition, the national survey explores a range of factors related to health, health behaviours and study ability, such as social relationships, studying and subsistence. The USHS 2016 report includes the complete questionnaire. It comprises several validated questionnaires as subsets and other self-standing items [27]. The questionnaire was in Finnish and Swedish (official languages in Finland). Participation in the survey is totally voluntary. We collected the data used here anonymously from the most recent survey carried out in 2016 with the permission of the FSHS authorities and the Ethics Committee of the University of Turku [27].

### Participants

The target population comprised Finnish undergraduate students under 35 years studying in Finnish universities, namely academic universities (Univ) and universities of applied sciences (UAS). This study included 14 out of 15 Univ and 24 out of 26 UAS, inclusion and exclusion criteria for the study and handling of the missing data are described in detail in the USHS report [27]. The study population comprised of 10 000 students (Univ: 4996, UAS: 5004) who received an initial invitation followed by five reminders (all were sent by email, except for the third, which was sent by mail). FSHS anonymised the participants by deleting personal data and adding ID numbers before the researchers had access to it. No incentives for answering were used. Participants with unspecified gender were excluded from the study. Statistical procedures (including post-stratification) were used to count weights for men and women separately for the educational sectors [27]. After weighting adjustments, the sample comprised 47% men and 53% women, representing well the target population.

### Dependent variable

The question “Do you feel scared about dental care?” enquired about the outcome variable of the study, dental fear with three answer options: “Not at all”, “Somewhat” and “Very”. We considered high dental fear to have the most critical impacts on dental health, failure to seek dental care and early termination of treatment plans. We therefore combined the two former answers options (“Not at all” and “Somewhat”) as the reference group for the data analyses. The question used refers directly to ‘dental care’ and can be considered as a measure of dental fear [28]. Previous studies in the Nordic countries have confirmed the validity and reliability of assessing dental fear with a single question [29–31].

### Independent variables

The questionnaire (Additional file 1) included SCOFF, the most widely used screening test for eating disorders [32]. It consists of five short questions on the main aspects of EDs (i.e. self-induced vomiting, losing control on amounts of servings, prominent recent weight loss, the perception of being fat and whether food dominates the respondent’s life). The questions were presented in the original order in closed Yes/No format; we considered two or more “Yes” answers a positive result (SCOFF positive), suggesting a high probability of having an ED [21]. A very recent systematic review and meta-analysis of 25 validation studies of SCOFF, reported a pooled sensitivity and specificity of 86% and 83%, respectively [32]. Validations of the Finnish version of SCOFF have found 78% sensitivity and 87% specificity, suggesting its usefulness in high-risk populations, such as adolescents and university students [33].

We used the self-reported height and weight to calculate BMI (weight (Kg) divided by the square of height (m2)). We considered BMI of < 15 or > 50 as exclusion criteria, but after examining the BMI scores, no such outliers were found. We classified the results as underweight (BMI < 18.5), normal weight (BMI 18.5–24.9), overweight (BMI 25–29.9) and obese and extremely obese (BMI ≥ 30). Self-reported height and weight has been shown to be a reliable measure of BMI [34–36].

We assessed the participants’ perceived state of mental well-being with the question “How would you describe your current state of mental well-being (e.g. mental balance)?” with the answer options “Very poor”, “Poor”, “Moderate”, “Good” and “Very good” (see Appendix). We later grouped these answer options into two categories: “Poor or moderate” which included the options “Very poor”, “Poor”, “Moderate”) and “Good or Very good” (which included the options “Good” and “Very good”). [31] This question about mental well-being was first used in the USHS 2012. Since the question directly refers to “mental balance”, it can be considered as a measure of perceived mental well-being [28].

We categorized age into three groups: 19–24, 25–30 and 31–35 years. This was done because age groups are different. The youngest age group e.g. has entered the Univ or UAS directly of shortly after graduating from college and they have shorter time from the onset of possible EDs.

In the questionnaire, a single question enquired about attitude towards food (“Is your attitude towards food normal?”) with three answer options: “Yes”, “No” and “I don’t know”. We interpreted the last option as avoiding answering and combined the latter two options in the analysis to make “normal attitude towards food” as the reference group. This question was included in this study as a general indicator of attitude towards food and was not use in the logistic regression analyses. (Students with not normal attitude towards food were more likely to report high dental fear in our previous study [11].)

The question “Has a doctor or psychologist diagnosed any permanent, long-term or frequently recurring illness, health problem or trauma that has caused you symptoms, or required treatment over the past 12 months (e.g. Anorexia, Bulimia or other EDs)?” assessed diagnosed EDs; the answer options were “Yes” or “No”.

### Statistical analyses

We used cross-tabulations to assess bivariate associations between dental fear and, BMI, SCOFF status, attitude towards food, diagnosed eating disorders (during last 12 months), age, gender and educational sector (Univ or UAS). The chi-squared test served to evaluate the statistical differences of the bivariate associations. After checking collinearity, we performed logistic regression analyses to test our hypotheses. Dental fear was the dependent variable and BMI, SCOFF status, age and educational sector were covariates. (Due to the low number of ED diagnosis this variable was not included in the logistic regression analyses.) We entered perceived mental well-being into the final model along with all other covariates to eliminate any possible confounding effect. We performed modelling separately, but with the same approach, for both genders. We considered values of p < 0.05 statistically significant. Weighting adjustments in the analyses served to correct for the underrepresentation of men in the data [27]. We used IBM SPSS Statistics for Windows, Version 25 for all analyses.

## Results

A total of 3110 students participated in this study. Participation was higher among women than among men (39% and 22%, respectively) and to correct this weighting adjustments were used. In twenty cases the respondents had not specified their gender as male or female and they were excluded from the study. The overall response rate was 31% (n = 3090, Univ 39%, UAS 25%).

Table 1 shows the participants’ background factors and distribution by gender of the variables studied. Most of the participants (91.1%) belonged to 19–30 years age group, and more than half (59.4%) attended academic universities. About one fifth of students reported abnormal attitude towards food (‘No’ and ‘I don’t know’ alternatives comprised 8.1% and 11.2% of the responses, respectively). Females reported abnormal attitude to food more often than did males (26.4% vs. 11.4%, p < 0.001). Overall, 9.2% of the students scored positive on SCOFF. More females than males had a positive SCOFF status (≥ 2), but not normal weight was reported more often in males than in females (both p < 0.001). High dental fear was more often reported in females than in males (11.2% vs. 3.8%, p < 0.001), accounting for 7.8% of all participants. Altogether 39 students reported a diagnosed eating disorder over the past 12 months and 53.8% of them scored positive in the SCOFF (data not presented).

**Table 1:** Age, educational sector, dental fear, SCOFF status, BMI, mental well-being, attitude towards food and diagnosed eating disorders of the study population (n = 3090) by gender

	All n (%)	Men n (%)	Women n (%)	*p* value^§^
Age (2877)				< 0.001
19–24	1444 (50.2)	634 (46.2)	810 (53.9)	
25–30	1178 (40.9)	623 (45.4)	555 (36.9)	
31–35	255 (8.9)	116 (8.4)	139 (9.2)	
Educational sector (3077)				0.440
Univ	1829 (59.4)	852 (58.7)	977 (60.1)	
UAS	1248 (40.6)	599 (41.3)	649 (39.9)	
Dental fear (3013)				< 0.001
No or low dental fear	2779 (92.2)	1352 (96.2)	1427 (88.8)	
High dental fear	234 (7.8)	54 (3.8)	180 (11.2)	
SCOFF status* (3003)				< 0.001
Negative (< 2)	2728 (90.8)	1372 (96.4)	1356 (85.8)	
Positive (≥ 2)	275 (9.2)	51 (3.6)	224 (14.2)	
BMI^†^ (3006)				< 0.001
< 18.5 (underweight)	61 (2.0)	16 (1.1)	45 (2.8)	
18.5–24.9 (normal weight)	2031 (67.6)	901 (63.3)	1130 (71.4)	
25–29.9 (overweight)	718 (23.9)	429 (30.1)	289 (18.3)	
≥ 30 (obese and extremely obese)	196 (6.5)	77 (5.4)	119 (7.5)	
Mental well-being				
Poor or moderate	1060 (34.6)	495 (34.2)	564 (35.0)	0.634
Good or very good	2000 (65.4)	952 (65.8)	1048 (65.0)	
Normal attitude towards food (3037)				< 0.001
Yes	2451 (80.7)	1273 (88.6)	1178 (73.6)	
No/I don’t know	586 (19.3)	164 (11.4)	422 (26.4)	
Diagnosed eating disorders				
Anorexia (2454)	11 (0.4)	1 (0.1)	10 (0.8)	0.010
Bulimia (2426)	14 (0.6)	2 (0.2)	12 (1.0)	0.010
Other (2415)	27 (1.1)	3 (0.3)	24 (1.9)	< 0.001

*Univ* Academic universities, *UAS* Universities of applied sciences

^§^Chi-square test

^†^BMI: Body mass index was calculated from self-reported height and weight

*SCOFF (sick, control, one stone, fat, food) scores 2 or more out of five were considered positive

Table 2 presents the distribution of background and study factors by SCOFF status and by dental fear. The SCOFF-positive students reported abnormal attitudes towards food, abnormal weight, and higher dental fear (p < 0.001) more often than did SCOFF-negative students. Students who did not report a normal attitude towards food or were overweight/underweight also more frequently reported high dental fear (p < 0.001). A positive SCOFF score was associated with high dental fear (p < 0.001).

**Table 2:** Age, gender, educational sector, dental fear, SCOFF status, BMI, mental well-being, attitude towards food of the study population (n = 3003) by SCOFF and by dental fear

	SCOFF			Dental fear		
	All n	SCOFF positive^*^ n (%)	*p-value*^*§*^	All n	High n (%)	*p-value*^*§*^
Age	**2882**			**2834**		0.097
19–24	1407	125 (8.9)		1310	112 (7.9)	
25–30	1145	114 (10.0)		1066	92 (7.9)	
31–35	250	16 (6.4)		254	30 (11.8)	
Gender	**3003**		< 0.001	**3013**		< 0.001
Men	1423	51 (3.6)		1406	54 (3.8)	
Women	1580	224 (14.2)		1607	180 (11.2)	
Educational sector	**3003**		0.033	**3014**		0.003
Univ	1775	146 (8.2)		1807	119 (6.6)	
UAS	1228	129 (10.5)		1207	115 (9.5)	
Dental fear	**2943**		< 0.001	–	–	
No or low dental fear	2715	229 (8.4)		–	–	
High dental fear	228	39 (17.1)		–	–	
SCOFF status	–	–		**2943**		< 0.001
Negative (< 2)	–	–		2675	189 (7.0)	
Positive (≥ 2)	–	–		268	39 (14.5)	
BMI^†^	**2955**		< 0.001	**2945**		< 0.001
< 18.5 (underweight)	62	9 (14.5)		62	6 (9.7)	
18.5–24.9 (normal weight)	1996	148 (7.5)		1978	137 (6.9)	
25–29.9 (overweight)	705	77 (10.9)		712	55 (7.7)	
≥ 30 (obese or extremely obese)	192	36 (18.8)		193	31 (16.0)	
Mental well-being	**2990**		< 0.001	**2765**		< 0.001
Poor or moderate	1035	165 (15.9)		922	110 (10.7)	
Good or very good	1935	109 (5.6)		1843	122 (6.2)	
Normal attitude towards food	**2987**		< 0.001	**2975**		< 0.001
Yes	2412	64 (2.6)		2399	157 (6.5)	
No/I don’t know	575	209 (36.3)		576	75 (13.0)	

*Univ* Academic universities, *UAS* Universities of applied sciences

^§^Chi-square test

*SCOFF (sick, control, one stone, fat, food) scores 2 or more out of five were considered positive

^†^BMI: Body mass index was calculated from self-reported height and weight

In the gender-specific logistic regression analyses, after controlling for age (Table 3), educational sector was associated significantly with high dental fear: female students from UAS experienced high dental fear more often than did their counterparts from academic universities (p < 0.001). Adding SCOFF status and BMI to the model (Model 2) revealed that overweight males were more likely than others to report high dental fear (OR = 2.4; CI 1.3–4.4). SCOFF-positive female students experienced high dental fear more often than did other females (OR = 1.6; CI 1.0–2.4). (Adding SCOFF status and BMI in a stepwise approach yielded no change in outcome, data not shown).

**Table 3:** Stepwise gender-specific results of logistic regression analyses (n = 3090^*^), dental fear being the dependent variable (high fear = 1)

	Model 1^a^			Model 2^b^			Model 3^c^		
	OR	95% CI	*p*	OR	95% CI	*p*	OR	95% CI	*p*
Women									
Educational sector	1.8	1.3–2.4	< 0.001	1.8	1.3–2.5	0.001	1.8	1.3–2.5	< 0.001
SCOFF				1.6	1.0–2.4	0.043	1.3	0.8–2.0	0.318
BMI^†^									
Underweight				0.9	0.3–2.4	0.779	0.8	0.3–2.2	0.653
Overweight				1.3	0.9–1.9	0.137	1.2	0.8–2.0	0.318
Mental well-being							2.1	1.4–2.9	< 0.001
Men									
Educational sector	1.1	0.6–2.0	0.768	1.0	0.6–1.8	0.969	1.0	0.6–1.8	0.987
SCOFF				1.8	0.6–5.8	0.305	1.6	0.5–5.3	0.404
BMI^†^									
Underweight				2.4	0.4–16.0	0.368	2.4	0.4–16.2	0.357
Overweight				2.4	1.3–4.4	0.003	2.4	1.3–4.3	0.004
Mental well-being							1.7	0.9–3.0	0.092

Reference groups = academic university students, SCOFF negative (score < 2), normal weight (18.5–24.9) and good or very good mental well-being

*OR* odds ratio, *95% CI* 95% confidence interval, *SCOFF* Sick, control, one stone, fat, food

^†^BMI: Body mass index (calculated from self-reported height and weight)

*Adjusted for underrepresentation of men

^a^Model 1 adjusted for age; ^b^Model 2 adds SCOFF status and BMI; ^c^Model 3 adds mental well-being

Adding perceived mental well-being to the previous models (Model 3) showed that overweight among the males still significantly associated with high dental fear (OR = 2.4; CI 1.3–4.3) (Table 3). Mental well-being associated significantly with high dental fear in females; students who reported very poor to moderate mental well-being were more likely to report high dental fear than were those with good or very good mental well-being status (p < 0.001), but SCOFF status was no longer significantly associated with dental fear.

## Discussion

This comprehensive study endorsed our hypothesis that students with an abnormal BMI are more likely to experience high dental fear. Overweight was significantly associated with high dental fear among males, whereas educational sector and mental well-being were significant determinants of high dental fear among females. Female respondents and students of UAS reported high dental fear more often.

In this study, the prevalence of high dental fear was significantly lower among students with normal BMI. In the logistic regression analyses, after controlling for age, educational sector, SCOFF status and mental well-being, we observed that overweight or obese males (BMI ≥ 25) were more likely than normal weight males to report high dental fear. There are only few studies investigating the association between dental fear and obesity, and the findings are inconsistent. One study found that obese females reported dental fear more often than others, while another research showed no significant difference between normal weight and overweight participants [25, 26]. Our finding that overweight males were more likely to have high dental fear than normal weight males is in concordance with findings of a recent systematic review indicating that anxiety in general occurs more frequently in obese/overweight people compared to normal weight persons [37].

We found an association between SCOFF status and dental fear, SCOFF-positive students reported high dental fear twice as often as SCOFF-negative students. Among females, when controlling for age, educational sector and BMI, those with positive SCOFF score were more likely to have high dental fear than those with negative SCOFF score, but this association was no longer significant after adding mental well-being to the model. Thus, the second part of our hypothesis was only partly true. Among males probably the low number of males with high dental fear and positive SCOFF-status (n = 54, n = 51, respectively) was behind the non-significant association between dental fear and SCOFF-status. The high impact of mental health on dental fear may have attenuated the observed association in females. However, the association between SCOFF status and dental fear found in females in the model controlled for age, educational sector and BMI, can still be clinically important. Dentists may see the early signs of ED’s and may have difficulties treating patients with high dental fear together other psychological problems (like EDs). Patients with high dental fear and EDs could benefit of team work of dentists and psychologists [38].

Although a few previous studies have reported high levels of anxiety among patients with EDs in dental settings [14], to the best of our knowledge, this is the first study to investigate the association between SCOFF status and dental fear. Our results show that about one in ten Finnish students scored positive on SCOFF. A recent systematic review and meta-analysis of 19 studies on student populations reported a 10.4% pooled prevalence of being at risk for EDs [39]. In studies limited to female participants only, the prevalence was as high as 38.2% [22]. The higher proportion of SCOFF-positive females in our study is in line with the current literature [13, 24].

Studies have well documented significant and positive correlation between SCOFF and other ED tests, and reported an association between SCOFF and BMI [22, 23]. In this study, only 39 students reported being diagnosed with EDs over the past 12 months, 53.8% of whom scored positive in the SCOFF, however the Finnish version of SCOFF has been validated to have 78% sensitivity [33]. In this study we expected higher percentage of SCOFF positives among those with ED diagnosis. One reason for the observed low percentage might be attributed to the fact that some of the students had received appropriate treatments to their EDs and then recovered, and therefore scored negative in the SCOFF. In addition, the high number of missing responses regarding EDs might be due to skipping sensitive questions to hide ED history. Furthermore, atypical EDs are fairly common among university students [40] and this could have affected the association between SCOFF and ED diagnosis. In general, health care system’s ability to identify EDs is limited and SCOFF can find symptoms of EDs not fulfilling the diagnostic criteria.

The connection between dental fear, BMI, EDs and problems of mental well-being may result from common vulnerability factors shared by psychological disorders [8]. For most people with dental fear, exogenous components, such as treatment or vicarious experiences [41], may be more important than psychopathology in the development of dental fear. However, among others, endogenous aetiology of dental fear may partially explain dental fear. The endogenous aetiology includes psychological vulnerability to anxiety disorders and other psychological problems [7, 8, 42]. Thus, the same students may have a constitutional vulnerability to developing, anxiety disorders (e.g. dental fear) and other problems of mental well-being (e.g. EDs) [6–8].

We collected the notably large data set used in the present study from an extensive national survey of the Finnish universities. To increase the students’ participation, the questionnaires were sent as emails followed by five reminder invitations. On the web, responders can skip some questions as they are not in a face-to-face interview, but studies report more missing values in paper-based surveys than in web-based ones [43]. Reports indicate that both designs have similar levels of selection bias [44]. The paper-based questionnaires can have slightly higher response rates, but web-based questionnaires are less expensive to administer, making them ideal for large-scale enquiries. The response rate of this study was in line with other web-based surveys [43–45]. Women participate in studies more often than men do and this was also the case in our study. In this study weighting adjustment (methodological approach previously proved successful [2, 3, 9–11, 31]), was used to compensate for the underrepresentation of men. The participants of this study represented well the target population for age, study field, faculty and educational sector, when comparing with the national statistics 2015 published by the Education Statistics Finland [46]. Additionally, the health and health habit findings of the USHS 2016 are comparable to those of previous USHS studies [27], indicating no remarkable downward or upward trend, which also suggests that the composition of the participants has not changed considerably.

Single questions have been reported valid and reliable in measuring dental fear, when comparing to multi item questionnaires [29–31]. Even though clinical studies more often use multi-item questionnaires, single questions are easier and faster to answer for screening purposes. Adults are afraid of dental treatment and invasive stimuli have been reported as the most anxiety provoking [47]. In this study, we used a single question, which directly refer to ‘dental care’; this question can be considered as a measure of dental fear [28]. Additionally, the question used in this study has given similar prevalence of dental fear as the questions referring to visiting a dentist [2, 3, 11]. Furthermore, enquiring dental fear out of a clinical setting, results in a more substantial estimation of and more comprehensive participation by the study population. This in turn reduces the risk for selection bias.

We calculated BMI using self-reported height and weight, which may be subject to subtle random errors. However, young university students are relatively aware of their body size and shape, and self-reported height and weight has been shown to be a reliable measure of BMI and predictor of obesity-associated health-risks [34–36]. This supports our belief that the data represents the accurate values. Because the survey focused university students, it did not include those who were not studying in academic universities or UAS. Thus, the results cannot be generalised to all young Finnish adults. In addition, due to the cross-sectional nature of the study, causal interpretation is not possible.

## Conclusions

Among Finnish university students BMI among males and problems of mental well-being among females were positively associated with high dental fear. The results of this study support possible common vulnerability factors that dental fear and other psychological disorders may share.

## Supplementary Information

**Additional file 1**. USHS 2016 questions used in this study.

## Data Availability

The data are available in the Finnish Social Science Data Archive where registered users can download data online according to the conditions set for this data. http://urn.fi/urn:nbn:fi:fsd:T-FSD3224

## Abbreviations

ED: Eating disorder
BMI: Body mass index
SCOFF: Sick, control, one stone, fat, food
Univ: Academic universities
UAS: Universities of applied sciences
USHS: University Student Health Survey
FSHS: Finnish Student Health Service
AN: Anorexia nervosa
BN: Bulimia Nervosa
OR: Odds ratio
CI: Confidence interval
