# Supplementary material for: Association between dental fear and eating disorders and Body Mass Index among Finnish university students: a national survey
Source: BMC Oral Health. 2021 Mar 4;21:93. doi: 10.1186/s12903-021-01449-8 (PMC7934505; doi:10.1186/s12903-021-01449-8)
Supplement: Supplementary file 1 — Additional file 1. USHS 2016 questions used in this study. [file 12903_2021_1449_MOESM1_ESM.pdf]

Supplementary file 1

USHS 2016 questions used in this study

1. Age ____ years

2. Gender
1. Male
2. Female
3. Other

3. Your height |___|___|___| cm

4. Your weight |___|___|___| kg

5a. In which university do you study?
UNIVERSITIES
1 Aalto University
2 University of Helsinki
3 University of Eastern Finland
4 University of Jyväskylä
5 Finnish Academy of Fine Arts
6 University of Lapland
7 Lappeenranta University of Technology
8 University of Oulu
9 Sibelius Academy
10 Hanken School of Economics
11 Tampere University of Technology
12 University of Tampere
13 Theatre Academy
14 University of Turku
15 University of Vaasa
16 Åbo Akademi University

5b. In which university do you study?
UNIVERSITIES OF APPLIED SCIENCES (=UAS)
1 Arcada UAS
2 Diaconia UAS (Diak)
3 Haaga-Helia UAS
4 Humak UAS
5 Häme UAS (HAMK)
6 Jyväskylä UAS (JAMK)
7 Kajaani UAS
8 Keski-Pohjanmaan UAS
9 Kymenlaakso UAS
10 Lapin UAS
11 Lahti UAS
12 Laurea UAS
13 Metropolia UAS

14 Mikkeli UAS
15 Oulu UAS
16 North Karelia UAS
17 Saimaa UAS
18 Satakunta UAS
19 Savonia UAS
20 Seinäjoki UAS
21 Tampere UAS
22 Turku UAS
23 Vaasa UAS (VAMK)
24 Yrkeshögskolan Novia UAS

6. Do you feel scared about dental care?

1. Not at all
2. Somewhat
3. Very much

7. The SCOFF questionnaire

1. Do you make yourself sick because you feel uncomfortably full?	1. Yes	2. No
2. Do you worry that you have lost control over how much you eat?	1. Yes	2. No
3. Have you recently lost more than six kilograms in a 3-month period?	1. Yes	2. No
4. Do you believe yourself to be fat when others say you are too thin?	1. Yes	2. No
5. Would you say that food dominates your life?	1. Yes	2. No

8. How would you describe your current state of mental well-being (e.g. mental balance)?

1. Very poor
2. Poor
3. Moderate
4. Good
5. Very good

9. Is your attitude to food normal?

1. No
2. Yes
3. I don't know

10. Has a doctor or psychologist diagnosed over the past year (12 months)?

Eating disorder, what kind?

1. Anorexia	1. Yes	2. No
2. Bulimia	1. Yes	2. No
3. Other	1. Yes	2. No
